# Supplementary material for: Exploring the pre-instruction gender gap in physics
Source: PLoS One. 2022 Jul 14;17(7):e0271184. doi: 10.1371/journal.pone.0271184 (PMC9282597; doi:10.1371/journal.pone.0271184)
Supplement: Attachment — Submitted filename: referee_report.pdf [file pone.0271184.s001.pdf]

Referee Report: Exploring the pre-instruction gender gap in physics

1 Summary

This is an interesting paper on the source of gender gaps in exam scores in an introductory physics course at university. The authors administer a test of physics-specific incoming preparedness (FMCE test) based on high school inputs to a sample of students in the very first class of an introductory physics course. They also survey the students to collect measures of social-psychological constructs such as test anxiety, sense of belonging, "science identity" and interest in physics. They model (using linear regression and structural equation modeling) the final exam scores of these students as a function of gender, FMCE test scores and social-psychological measures, and find that the entire gender gap in exam scores can be explained by a gender gap in the FMCE. They argue that both gender gaps in the FMCE and exam scores could arise out of timed-test anxiety, and find no evidence of gender gaps in non-timed tests.

I believe this paper makes some very interesting arguments but the empirical work could be made more transparent and clear, and the final claim on the impact of timed test anxiety could be further substantiated. Detailed comments and suggestions follow:

2 Comments

1. The authors set out to show all their results using both linear regression models and structural equation modelling (SEM). However, the two sets of models that they estimate are not directly comparable to one another because of the choice of mediating variables in either case. I suggest that the authors make these results more easily comparable in the following ways:

   (a) The SEM in Figure 3 has no counterpart in terms of a linear regression model. Before Table 2, the authors could include a table with the results of the estimation of a linear regression model of FMCE scores as a function of a) gender, b) HS courses and gender, and c) social-psychological constructs (test anxiety, science identity, interest, belonging), HS courses and gender (including interactions, if any). The current Table 2 and Table 3 could even be combined into a single table if the authors are struggling to accommodate an additional table into this short paper.

(b) The current Table 2, which is the linear regression counterpart to the SEM in Figure 4, should include the results from a model which, in addition to controlling for gender and FMCE, also includes the four social-psychological construct measures (test anxiety, science identity, interest, belonging). Indeed, the "example regression model" on page 7 does include text anxiety as an explanatory variable, but this does not show up in the results table. Table 2 should also include a model with both FMCE and the number of HS courses, since the SEM in Figure 4 allows for both to have direct effects on exam grades.

(c) The linear regression model in Table 4 has no counterpart in terms of an SEM. The authors should provide an SEM or briefly explain why this has been omitted, particularly since they state in the methods section that their preferred model is the SEM. Again, for a direct comparison with the effects of gender on the timed test, the estimated linear regression models in Table 4 should be identical to the ones in Table 2 (and should include social-psychological constructs).

2. There is no mention of the number of observations at any point in the paper. This should be included in all tables and figures.

3. In Table 3 as well as in the accompanying text, there is no explanation of the variables AP-1 and AP-C. The accompanying text only mentions one variable: whether a student took an AP mechanics course. This needs to be clarified.

4. If, as the second explanation in the Discussion section states, the only reason for the gender gap in the FMCE is test anxiety, then it is not clear why the FMCE would have a causal impact on the the gender gap in exam scores in college: the gender gap should be mediated through differences in test anxiety, rather than through differences in the level of preparedness. In the SEM, test anxiety does not appear to have a direct impact on exam scores (and this can be examined in the regression model framework as well, as I suggest above). This suggests that the FMCE scores capture differential preparedness by gender, and the FMCE predicts final exam scores (over and above test anxiety), which together explain a gender gap in exam results.

5. It is hard to draw conclusions about the first homework assignment without knowing more about what was assessed on this assignment and how comparable it is to the FMCE. The authors could discuss this in a little more detail. Perhaps the homework is simply not predictive of the exam score in the same way that the FMCE is: this could be assessed by modelling the final exam score as a function of gender, homework 1 score, and social-psychological construct measures. Another concern is that the homework is presumably graded, while the FMCE is not. Prior research has shown that women care more about their grades at university than men do, which could be one reason why they close the the gender gap (relative to the FMCE score). If the authors have access to all homework scores across the entire semester, one interesting extension to

this paper could be to model gender gaps in each homework score all the way up to the final exam score. This could shed light on when gender gaps in performance emerge, and how large the gaps in the final exam score are, relative to all the preceding timed and non-timed homework assignments. If learning by female students is negatively affected by an adverse classroom environment at university, one may see gender gaps in scores widen over the course of the semester.

2.1 Minor Comments

1. While I understand that it is beyond the scope of a short paper to discuss in detail all the different social-psychological construct measures that are used here, a short sentence or two discussing what each measure purports to capture will be very useful for the reader, particularly of a general-interest journal like this one. Perhaps this could be included in Table 1 in an additional column.

2. The authors mention the "TFI" measure of goodness-of-fit for the SEM; do they mean TLI?

3. In the first instance, acronyms like URM should be fully stated, and thereafter referred to by the abbreviation.

4. There are several typos in the text. The authors should thoroughly and carefully proofread it.
