# Supplementary material for: Exploring the pre-instruction gender gap in physics
Source: PLoS One. 2022 Jul 14;17(7):e0271184. doi: 10.1371/journal.pone.0271184 (PMC9282597; doi:10.1371/journal.pone.0271184)
Supplement: Attachment — Submitted filename: referee_report2.pdf [file pone.0271184.s004.pdf]

Referee Report Round 2: Exploring the pre-instruction gender gap in physics

I thank the authors for responding to the comments and addressing most concerns. I believe this paper provides interesting insights on an important dimension of the gender gap in STEM. Minor comments and suggestions follow:

1. Table 3 heading should read “FMCE scores” rather than “exam scores” for clarity.

2. There remain many typos in the text, including in the new sections that have been added. This detracts from the readability of the manuscript. Please do your best to address them. I mention a few examples below from the newly added text:

   - Second equation on page 8 should have “Incoming Preparation” as the dependent variable, not “Incoming Prepartion”.
   - You switch between Roman and Arabic numbering of your tables as in “Table II” and “Table III” on page 11 and “Table 4” and “Table 6” elsewhere. These should be consistent.
   - From the new paragraph added to the conclusion (page 24): “the lack of gender gap in physics course taking in high school” should read “the lack of a gender gap in physics courses taken in high school”; “so we believe are likely more general” should read “so we believe these results are likely to be more general”; “It is unclear the extent to which test anxiety plays a role in assessment demographic gaps” should read “It is unclear the extent to which test anxiety plays a role in demographic gaps in assessment”.
