# Supplementary material for: Exploring the pre-instruction gender gap in physics
Source: PLoS One. 2022 Jul 14;17(7):e0271184. doi: 10.1371/journal.pone.0271184 (PMC9282597; doi:10.1371/journal.pone.0271184)
Supplement: Attachment — Submitted filename: RefereeResponse_v2.docx [file pone.0271184.s002.docx]

We thank the editor and referees for their thoughtful feedback. The proposed revisions will substantially improve the manuscript.

**Referee 1**

2. 1. A) The SEM in Figure 3 has no counterpart in terms of a linear regression model. Before Table 2, the authors could include a table with the results of the estimation of a linear regression model of FMCE scores as a function of a) gender, b) HS courses and gender, and c) social-psychological constructs (test anxiety, science identity, interest, belonging), HS courses and gender (including interactions, if any).

*We have included the recommended table to complement Figure 3. The results clearly show that regression and SEM give the same findings.*

2. 1. B) The current Table 2, which is the linear regression counterpart to the SEM in Figure 4, should include the results from a model which, in addition to controlling for gender and FMCE, also includes the four social- psychological construct measures

*We have chosen to make this a separate table (Table 5), but have included the requested regressions. These again show that all of the exam performance is mediated by FMCE pre-score.*

2.1.C ) The linear regression model in Table 4 has no counterpart in terms of an SEM. The authors should provide an SEM or briefly explain why this has been omitted, particularly since they state in the methods section that their preferred model is the SEM.

*We have clarified that there is no corresponding SEM for this model because we did not have social psychological variables.*

2.2) There is no mention of the number of observations at any point in the paper.

*We have included the number of observations in each table.*

2.3) In Table 3 as well as in the accompanying text, there is no explanation of the variables AP-1 and AP-C.

*We have explained this further “The size of the gender gap also remains the same when controlling for whether a student had taken an AP mechanics course (AP Physics 1 is an algebra based college-level physics course, AP Physics C is the calculus based version of the same course)."*

2.4) If, as the second explanation in the Discussion section states, the only reason for the gender gap in the FMCE is test anxiety, then it is not clear why the FMCE would have a causal impact on the the gender gap in exam scores in college: the gender gap should be mediated through differences in test anxiety, rather than through differences in the level of preparedness. In the SEM, test anxiety does not appear to have a direct impact on exam scores (and this can be examined in the regression model framework as well, as I suggest above). This suggests that the FMCE scores capture differential preparedness by gender, and the FMCE predicts final exam scores (over and above test anxiety), which together explain a gender gap in exam results.

*To address this point, we have added a paragraph to the discussion in page 21:* ***“******We see that the gender gaps in exam performance are entirely mediated by FMCE pre-scores, suggesting that any effects of test anxiety on final exam performance are captured by the timed nature of the FMCE. This would suggest that the stakes of the assessment do not seem to matter as much as the fact that the assessment is timed. This factor appears to explain a portion of the gender gap, but a substantial amount of the gender gap is left unexplained by variables we collected. Because we find no gap in Homework 1 scores, we hypothesize that some other social-psychological measure might explain the remainder of this gap. There are many aspects of student thinking for which we do not have validated psychometric scales. For example, math anxiety is considered to be a separate construct from test anxiety. In the future, we will collect math anxiety measures to see if this explains part of the gap, as math is an important part of physics preparation and the FMCE contains many graphical interpretation questions.”***

2.5) It is hard to draw conclusions about the first homework assignment without knowing more about what was assessed on this assignment and how comparable it is to the FMCE. The authors could discuss this in a little more detail.

*We have added a paragraph to the end of the results section in page 20:* ***“We note that HW1 covers much of the same content as the FMCE – basic 1D kinematics – thus it is notable that there is no gender gap on the assignment. Indeed, the assignment is still strongly predictive of final exam score (r = 0.26, p < 0.0001). This is not quite as strong as the correlation with FMCE score, likely because the FMCE covers a wider range of content and is thus more predictive of overall course performance. In prior work [3] we have seen similar patterns of the lack of gender gaps in measures that include homework scores. ”***

2.1.1) While I understand that it is beyond the scope of a short paper to discuss in detail all the different social-psychological construct measures that are used here, a short sentence or two discussing what each measure purports to capture will be very useful for the reader

*We have added a short sentence explaining what this construct measures to Table 1.*

2.1.2) The authors mention the “TFI” measure of goodness-of-fit for the SEM; do they mean TLI?

*We have changed TFI to TLI.*

2.1.3) In the first instance, acronyms like URM should be fully stated, and thereafter referred to by the abbreviation.

*We have defined URM in the beginning of page 4 as: “*non-white and non-Asian under-represented racial minority*“.*

2.1.4) In the first instance, acronyms like URM should be fully stated, and thereafter referred to by the abbreviation.

*We have had the manuscript proofread.*

**Referee 2.**

1) For international readers, it will be good to explain some of the terms specific to the US context, such as FMCE scores in the abstract, URM in introduction, SAT/ACT score in the introduction, R1 university in the data section, etc

*We have added more details about each of the terms identified by the reviewer.’*

2) It seems that RQ1 and RQ2 discussed in the theoretical framework have already been explored in the literature and the current paper is just replicating the findings for the sample considered here. This aspect should be explicitly mentioned when the research questions are stated at the end of the theoretical framework section.

*WE have noted this to the end of theoretical framework section in page 7* ***“Note that there are a number of studies that have already investigated RQ1 and RQ2. We repeat them for the current sample to provide more context to the results of RQ3.”***

3a) The sample size is not given anywhere in the paper. I suggest that the estimation sample size is given for each regression (below R-squared).

*We have added sample sizes to all tables.*

3b) There is no discussion on the external validity of these results. This could be addressed to some extent by discussing the nature of the sample -- what kind of population of students it represents.

*We have clarified that the results in Table 1 are likely due to the select sample size and added a paragraph to the conclusions:* ***“We encourage other researchers to perform similar analyses with different student populations. We expect that some of the results (the lack of gender gap in physics course taking in high school, for example) are due to the highly selective nature of the population. Some of the other findings such as the gender gaps in sense of belonging and test anxiety are consistent with other reports, and so we believe are likely more general. It is unclear the extent to which test anxiety plays a role in assessment demographic gaps. We suspect this is somewhat sensitive to the nature of the assessment and population as shown in previous works (Salehi et al., 2021), and thus may not be replicated for other assessment environments.”***

3c) The regression equations are not written accurately; if the error term is omitted, then it would mean that the explanatory variables included in the model perfectly predict the outcome, which is incorrect.

*We have included the error term.*

4)Since the main contribution of the paper relies on the measures described in Table 1, would it be possible to clarify these measures in the table itself, instead of mentioning the references only? That would make the paper self-sufficient and help the reader understand the measures directly from this paper.

*We have further defined these measures in Table 1.*
